# Adrenergic regulation of monocyte chemotactic protein 1 leads to enhanced macrophage recruitment and ovarian carcinoma growth

**DOI:** 10.18632/oncotarget.2887

**Published:** 2014-12-27

**Authors:** Guillermo N. Armaiz-Pena, Vianey Gonzalez-Villasana, Archana S. Nagaraja, Cristian Rodriguez-Aguayo, Nouara C. Sadaoui, Rebecca L. Stone, Koji Matsuo, Heather J. Dalton, Rebecca A. Previs, Nicholas B. Jennings, Piotr Dorniak, Jean M. Hansen, Jesusa M.G. Arevalo, Steve W. Cole, Susan K. Lutgendorf, Anil K. Sood, Gabriel Lopez-Berestein

**Affiliations:** ^1^ Department of Gynecologic Oncology and Reproductive Medicine, The University of Texas M. D. Anderson Cancer Center, Houston, Texas 77030, USA; ^2^ Department of Experimental Therapeutics, The University of Texas M. D. Anderson Cancer Center, Houston, Texas 77030, USA; ^3^ Department of Obstetrics and Gynecology, The John Hopkins University, Baltimore, MD 21287, USA; ^4^ Division of Gynecologic Oncology, Department of Obstetrics and Gynecology, University of Southern California, Los Angeles, CA 90089, USA; ^5^ Department of Medicine, Division of Hematology-Oncology, University of California, Los Angeles, CA 90095, USA; ^6^ Department of Psychology, Obstetrics and Urology, and Holden Comprehensive Cancer Center, University of Iowa, Iowa City, Iowa 52242, USA; ^7^ Department of Cancer Biology, The University of Texas M. D. Anderson Cancer Center, Houston, Texas 77030, USA; ^8^ Center for RNA Interference and Non-coding RNA, The University of Texas M. D. Anderson Cancer Center, Houston, Texas 77030, USA

**Keywords:** ovarian cancer, macrophages, monocytes, catecholamines, MCP1

## Abstract

Increased adrenergic signaling facilitates tumor progression, but the underlying mechanisms remain poorly understood. We examined factors responsible for stress-mediated effects on monocyte/macrophage recruitment into the tumor microenvironment, and the resultant effects on tumor growth. *In vitro*, MCP1 was significantly increased after catecholamine exposure, which was mediated by cAMP and PKA. Tumor samples from mice subjected to daily restraint stress had elevated MCP1 gene and protein levels, increased CD14+ cells, and increased infiltration of CD68+ cells. hMCP1 siRNA-DOPC nanoparticles significantly abrogated daily restraint stress-induced tumor growth and inhibited infiltration of CD68+ and F4/80+ cells. In ovarian cancer patients, elevated peripheral blood monocytes and tumoral macrophages were associated with worse overall survival. Collectively, we demonstrate that increased adrenergic signaling is associated with macrophage infiltration and mediated by tumor cell-derived MCP1 production.

## INTRODUCTION

Sustained adrenergic activation resulting from prolonged behavioral stress leads to increased tumor growth [1, 2]. Chronic increases in pro-tumoral catecholamines, norepinephrine and epinephrine, affect a broad array of processes involved in the progression and metastasis of various cancers [3]. Sympathetic nervous system activity directly influences the pathogenesis of ovarian carcinoma by protecting tumor cells from anoikis [4], promoting tumor cell invasion [5–7] and increasing tumor-associated angiogenesis [1, 8]. These data highlight the importance of the tumor microenvironment in these processes, but the underlying mechanisms require further analysis.

Tumor associated macrophages (TAMs) are key components of the tumor microenvironment [9] and reflect a continuum of maturation/differentiation of bone marrow derived monocyte precursors that adapt to the different microenvironments where they will eventually reside [10]. They differentiate from circulating blood monocytes recruited into the tumor microenvironment and are rapidly activated by tumor cells. Activated TAMs release cytokines, chemokines, enzymes, and pro-inflammatory factors that lead to further macrophage recruitment and increased tumor growth [11]. Recently, it has been reported that patients with higher levels of adrenergic signaling have higher counts of MMP-9-producing TAMs [12]. Additionally, in an orthotopic mouse model of breast cancer, restraint stress was shown to induce breast cancer metastasis, largely mediated by increased macrophage infiltration into the tumor [13]. While several studies have demonstrated that increased adrenergic signaling leads to enhanced macrophage recruitment into the tumor, the underlying mechanisms remain poorly understood. We describe an ADRB/PKA axis that mediates increased MCP1 levels resulting in enhanced macrophage infiltration and tumoral growth. Furthermore, we demonstrate that increased peripheral blood monocytes and TAMs are associated with advanced disease and decreased overall survival in ovarian cancer patients.

## RESULTS

### NE and Epi increase ovarian cancer cell derived-MCP1 production

To elucidate the mechanism used by tumor cells to induce monocyte/macrophage recruitment while undergoing sustained adrenergic signaling, we analyzed chemokine/cytokine changes using a cytokine/chemokine panel generated from SKOV3ip1 cells treated with norepinephrine or epinephrine. Several cytokines, such as fractalkine, IL6, IL8, MCP1 and VEGF were identified to be upregulated by both catecholamines (Supplementary Table 1), while other key players in the induction of monocyte/macrophage recruitment, such as CSF1, were not found to be elevated after NE treatment (Supplementary Table 1 and Supplementary Figure 1a). Hence, we decided to focus on MCP1 due of its known role in the induction of macrophage recruitment into the microenvironment.

**Figure 1:**
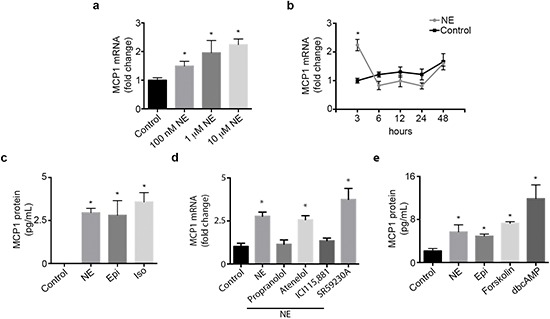
Catecholamines induce MCP1 expression **(a)** SKOV3ip1 cell MCP1 gene expression after treatment with several doses of NE for 3 hours. **(c)** SKOV3ip1 cell MCP1 gene expression after treatment with 10 μM NE for various time intervals. **(b)** MCP1 concentration in medium from SKOV31ip1 cells after treatment with 10 μM norepinephrine, 10 μM epinephrine and 10 μM isoproterenol for 3 hrs. **(d)** MCP1 gene expression after treatment with 10 μM propranolol, 10 μM atenelol, 1 μM ICI115,881 or 10 μM SR59230A followed by 10 μM norepinephrine for 3 hrs. **(e)** MCP1 concentration in medium from SKOV3ip1 cells after treatment with 10 μM norepinephrine, 10 μM epinephrine, 1 μM forskolin and 10 μM dbcAMP for 3 hrs. *P < 0.05.

We examined the expression pattern of MCP1 in an ovarian cancer cell panel. RT-PCR results show that MCP1 expression varied among all cell lines tested, with SKOV3ip1, A2780-CP20, SKOV3ip1-TR and 2774 cells being MCP1 positive (Supplementary Figure 1b). Next, we examined if norepinephrine or epinephrine stimulation could directly increase MCP1 production by ovarian cancer cells. To confirm that SKOV3ip1 cells respond to NE treatment, we determined cAMP levels by ELISA. cAMP levels were significantly increased at 15 and 30 min after NE treatment (Supplementary Figure 1c). A significant dose-dependent increase in MCP1 gene production was seen in SKOV3ip1 cells at 3 hours following NE treatment (Figure 1a). This gene increase peaked at 3 hours and subsequently returned to baseline (Figure 1b). To ascertain that gene stimulation was followed by protein production, we further analyzed supernatant obtained from SKOV3ip1 cells treated with NE or Epi. MCP1 protein secretion was significantly enhanced after treatment with both catecholamines (Figure 1c). This same effect was seen after treatment with isoproterenol, an ADRB agonist (Figure 1c). To determine which adrenergic receptor mediate NE-induced MCP-1 gene expression, we treated SKOV3ip1 cells with propranolol (non-specific beta-blocker), atenelol (ADRB1), ICI115,881 (ADRB2) or SR59230A (ADRB3) and added NE for 3 hrs. A significant increased in MCP1 gene expression was seen after NE exposure and this effect was abrogated by propranolol and ICI115,881 (Figure 1d). Since downstream ADRB signaling can be largely mediated by increased cAMP and PKA activity, we treated SKOV3ip1 cells with forskolin (cAMP agonist) or dbcAMP (PKA agonist). Results demonstrated that forskolin or dbcAMP could induce cancer cell-derived MCP1 production (Figure 1e). As a whole, these data suggest that catecholamines can induce MCP1 production through an ADRB/cAMP/PKA signaling axis.

### Restraint stress leads to increased tumor cell-derived MCP1 production, monocyte and macrophage recruitment

**Figure 2:**
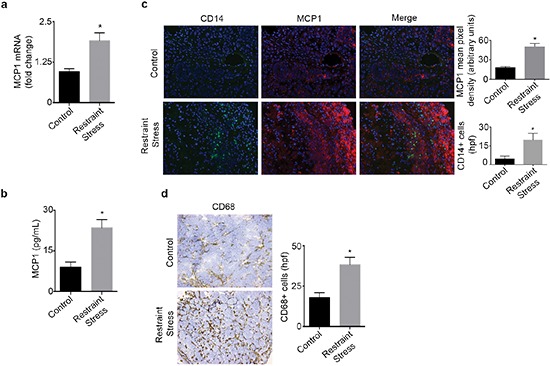
Daily restraint stress leads to increased MCP1 expression and infiltration of CD14+ and CD68+ cells **(a)** MCP1 mRNA or **(b)** protein levels in tumor samples from mice subjected to daily restraint stress. **(c)** Immunofluorescence analysis of tumor samples from mice subjected to daily restraint stress showing the presence of MCP1 and CD14+ cells. **(d)** Immunohistochemical analysis of tumor samples from mice subjected to daily restraint stress showing the presence of CD68+ cells. *P < 0.05.

We next investigated if increased adrenergic signaling could enhance tumor derived MCP1 production *in vivo*. To induce tumoral catecholamine levels, we used a well-characterized restraint stress model [1, 14]. Mice inoculated with SKOV3ip1 cells were subjected to daily restraint stress and tumor samples were analyzed for MCP1 gene and protein levels. After three weeks of restraint stress, a significant induction of MCP1 gene expression levels was observed (Figure 2a). In this same model, we observed a significant week-by-week increase in MCP1 gene expression, suggesting that MCP1 production is enhanced as the tumor progresses under sustained adrenergic signaling (Supplementary Figure 2a). This increase was significantly associated with elevated tumoral NE (Supplementary Figure 2b–2c). Additionally, tumor samples obtained from mice inoculated with 2774 cells and subjected to daily restraint stress exhibited a significant increase in MCP1 protein (Figure 2b). To determine if increased MCP1 production correlated with monocyte recruitment, we assessed samples for MCP1 and CD14 expression by immunofluorescence. This analysis revealed that restraint stress increased MCP1 production, and in areas with elevated MCP1 levels, CD14+ cells were observed (Figure 2c). These samples were also analyzed for the presence of CD68+ cells. We observed an increase in CD68+ cells in samples from mice subjected to daily restraint stress (Figure 2d).

### Restraint stress requires tumor-derived MCP1 to promote macrophage infiltration and tumor growth

**Figure 3:**
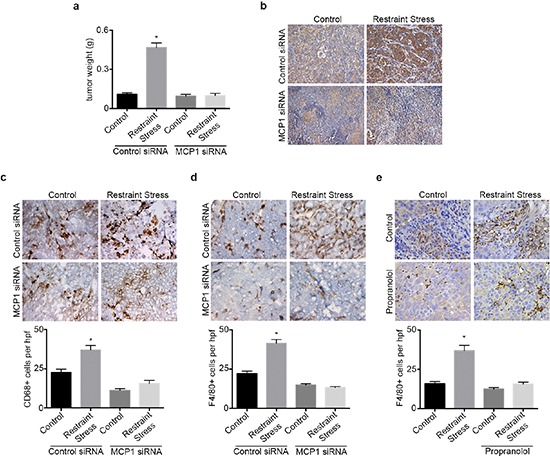
hMCP1 siRNA-DOPC nanoparticles abrogates restraint stress induced increase in tumor growth and macrophage recruitment **(a)** Tumor weight in restraint stress orthotopic SKOV3ip1 model treated with control or hMCP1 siRNA-DOPC nanoparticles. **(b)** MCP1 immunohistochemical analysis of tumor samples. **(c)** CD68 and **(d)** F4/80 immunohistochemical analysis of tumor samples. **(e)** F4/80 immunohistochemical analysis of tumor samples from mice subjected to restraint stress and propranolol. *P < 0.05.

We next investigated the role of MCP1 in the promotion of macrophage recruitment and tumor growth in response to sustained adrenergic signaling. To induce tumoral catecholamine levels, we used a well-characterized restraint stress model. First, we confirmed the efficacy of siRNA for silencing MCP1 in SKOV3ip1 cells (Supplementary Figure 3a). Subsequently, mice inoculated with SKOV3ip1 cells were subjected to daily restraint stress and treated with either control siRNA-DOPC or a validated hMCP1 siRNA-DOPC. In this model, restraint stress resulted in increased tumor growth, nodule counts and MCP1 levels, while hMCP1 siRNA-DOPC completely abrogated this effect (Figure 3a–3b and Supplementary Figure 3b). The increase in tumor growth in response to daily restraint stress was also accompanied by increased infiltration of CD68+ cells, but this effect was blocked by hMCP1 siRNA-DOPC (Figure 3c). Furthermore, restraint stress increased the amount of F4/80+ cells, while hMCP1 siRNA-DOPC abrogated these effects (Figure 3d). Next, we asked if propranolol could block restraint stress induced macrophage infiltration into the tumor. Results show that restraint stress increased the presence of F4/80+ cells, while propranolol blocked this effect (Figure 3e). These data point towards MCP1 as a key driver of restraint stress-induced macrophage recruitment ultimately leading to increased tumor growth.

### Elevated peripheral blood monocyte levels and tumoral macrophages are associated with poor outcome in patients with ovarian cancer

**Figure 4:**
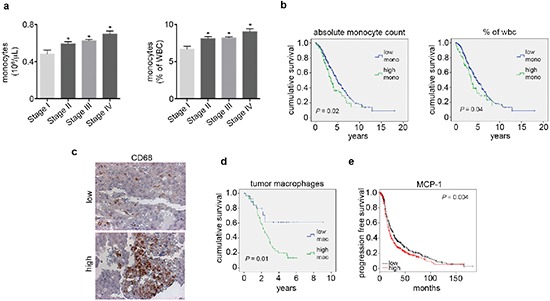
Elevated peripheral blood monocytes and tumoral macrophages correlate with poor outcome in ovarian cancer patients **(a)** Peripheral blood monocyte levels by ovarian cancer stage. **(b)** Kaplan-Meier curves of disease-specific survival in patients with epithelial ovarian carcinoma (*n* = 462) with high or low peripheral blood monocyte levels. **(c)** Representative picture of CD68 immunohistochemical analysis of patient tumor samples. **(d)** Kaplan-Meier curve of disease-specific survival in patients with epithelial ovarian carcinoma (*n* = 76) with high or low macrophage counts. **(e)** Kaplan-Meier curve of relapse free survival in patients with epithelial ovarian carcinoma (*n* = 1,171) with high or low MCP1 mRNA obtained from KM Plotter. *P < 0.05.

Next, we examined the levels of peripheral blood monocytes in a cohort of ovarian cancer patients (*n* = 462) and studied associations between clinical and pathological variables (Supplementary Table 2). Of the patients evaluated, 28% had elevated absolute peripheral blood monocyte counts (> 0.7 × 10^6^/μL) and 43% had high peripheral blood monocytes when measured as the percent of white blood cell content (> 8%). Elevated peripheral blood monocytes were significantly associated with increased ovarian cancer stage at the time of diagnosis and decreased survival (Figure 4a–4b and Supplementary Figure 4a–4b). A subset of these patients had tumor samples available for assessment of CD68+ cell infiltration (*n* = 76). Of the samples evaluated, 50% had elevated CD68+ counts (determined as > 15 per high power field; 20x). High counts of CD68+ cells were significantly associated with decreased overall survival (Figure 4c–4d). We carried out a multivariate analysis including CD68+ counts, stage, grade and extent of cytoreduction. In this analysis, elevated CD68+ cell count (*p* = 0.04) and suboptimal cytoreduction (*p* = 0.003) remained significantly associated with poor patient survival. Additionally, peripheral blood monocytes were significantly correlated with increased tumoral CD68+ cells (Supplementary Figure 4c). Furthermore, we looked for correlation between tumoral MCP1 gene levels and elevated CD68+ counts. Our data demonstrates that elevated MCP1 gene levels were correlated with higher CD68+ counts (Supplementary Figure 4d). We also studied the prognostic value of MCP1 mRNA expression using the Kaplan-Meier plotter (http://kmplot.com/analysis/index.php?p=service&cancer=ovar), which assesses the effect of 22,277 genes on survival in 1,171 ovarian cancer patients [15]. KM plotter analysis showed that elevated MCP1 mRNA expression had a negative impact on patients' progression free survival (Figure 4e).

## DISCUSSION

We demonstrate that increased adrenergic signaling leads to enhanced macrophage recruitment, which is mediated by tumor cell-derived MCP1. Clinically, elevated peripheral blood monocytes and tumoral macrophages were significantly associated with decreased survival in ovarian cancer patients. To the best of our knowledge, this is the first report to elucidate the underlying mechanism by which ovarian cancer cells, under sustained adrenergic signaling, recruit macrophages into the tumor microenvironment.

Previous work has shown that macrophage infiltration into the tumor microenvironment is chemokine and cytokine driven [9]. One key factor is MCP1, a potent monocyte/macrophage chemotactic agent [16]. MCP1 is produced by stromal components such as fibroblasts, endothelial cells, monocytes, and as shown in this study, tumor cells [9]. Chronic inflammation, observed in the tumor microenvironment, has been correlated with increased expression of cytokines, including MCP1 [17]. This increase has been associated with enhanced macrophage infiltration and tumor progression in breast cancer [17]. Previous reports have suggested that ovarian cancer cells are a major source of MCP1 and a key player in the recruitment of macrophages [18]. Recently, microarray analyses of tumors from ovarian cancer patients at greater biobehavioral risk (i.e., high depression and low social support) revealed an upregulation of MCP1 (reported as CCL2) gene expression. This elevated expression may be correlated to sustained tumoral adrenergic signaling. [19]. Another study demonstrated that tumor samples from ovarian cancer patients with greater biobehavioral risk had higher levels of MMP2+ and MMP9+ macrophage populations [12]. This increase in TAMs may be correlated with higher levels of tumoral adrenergic signaling that could facilitate TAM-induced tumor cell metastasis *via* increased MMPs release. These data suggest a role for MCP1 as a key mediator of adrenergically-induced macrophage infiltration and ovarian cancer growth.

These findings demonstrate another mechanism by which sustained adrenergic signaling induces tumor growth. Under this condition tumoral catecholamines stimulate ovarian cancer cells to secrete MCP1, which promotes macrophage infiltration. Once in the tumor microenvironment, TAMs mature, secrete pro-tumoral cytokines, and facilitate increased tumor growth. Future studies should examine the exact role of TAMs, how sustained adrenergic signaling affects their activity, and how they alter the tumor microenvironment. Our findings implicate MCP1 as another potential therapeutic target, which would inhibit tumor-promoting effects caused by increased adrenergic signaling.

## MATERIALS AND METHODS

### Reagents

Norepinephrine, epinephrine, isoproterenol and dibutyryl-cAMP, propranolol were purchased from Sigma-Aldrich (St. Louis, MO). Forskolin was purchased from Calbiochem (San Diego, CA). Atenelol, ICI115,881 and SR59230A were purchased from Tocris Biosciences (Minneapolis, MN).

### Cell lines and culture conditions

The derivation and source of the established ovarian cancer cell lines 2774 and SKOV3ip1 have been reported previously [1]. Cells were maintained in RPMI 1640 supplemented with 10% fetal bovine serum and 0.1% gentamicin sulfate (Gemini Bioproducts; Calabasas, CA). All cell lines were routinely screened for Mycoplasma species (Fisher; Itasca, IL). All experiments were performed with cell cultures at 70%–80% confluence.

### Enzyme-linked immunosorbent assay

MCP1 and CSF1 protein levels were determined by ELISA using the Quantikine Immunoassay kit (R&D Systems, Minneapolis, MN) or by Milliplex MAP analyses Millipore, MA) according to the manufacturer's protocol. Samples were assayed in duplicate, and data represent the mean concentration average over triplicate experiments.

### Cytokine assay

Supernatant from cultured ovarian cancer cells were stored at −20°C for batch analyses to measure cytokines. Supernatants were evaluated for cytokines/chemokines using the Milliplex MAP human cytokine/chemokine panel (Millipore, MA). Cytokine levels were measured in 50 μL of supernatant by Multiplex cytometric bead array (Multiplex) assay on a Luminex 100 Analyzer (Luminex Corp., Austin, Texas). The inter-variability for all inflammatory cytokines tested was less than 10%, indicating the highly reliability of the Multiplex-Luminex method of cytokine assay.

### Quantitative PCR

For mRNA quantification, ovarian cancer cell or tumor sample total RNA was extracted using RNeasy kit (Qiagen, Valencia, CA). Analysis of mRNA levels was performed on a 7500 Fast Real Time PCR system (Applied Biosystems) with SYBR Green-based real time PCR. Specific Primers for human MCP1 (forward)-5′-AGGTGACTGGGGCATTGAT-3′, (reverse)-5′-GCCTCCAGCATGAAAGTCTC-3′ were used. 18S was used as a housekeeping gene.

### siRNA preparation and treatment

Human MCP1-specific siRNA and Control siRNA was purchased from Sigma Genosys (The Woodlands, TX). For *in vitro* studies, cells were transfected with siRNA that was incorporated into Hiperfect transfection reagent (Qiagen, San Diego, CA) following the manufacturer's protocol. After 48 h, the experiments were carried out. For *in vivo* studies, siRNA was incorporated into the neutral liposome 1,2-dioleoyl-sn-glycero-3-phosphatidylcholine (DOPC) as previously described [20]. For each treatment, 3.5 μg of siRNA was reconstituted in 200 μL of phosphate buffered saline and administered by intravenous injection. The dosing schedule is described in the *in vivo* tumor model below.

### Chronic stress murine model

We obtained 8- to 12-week-old female athymic nude mice from the National Cancer Institute. All experiments were approved by the Institutional Animal Care and Use Committee at M. D. Anderson Cancer Center. To experimentally induce stress, we used a daily physical-restraint-stress model as previously described [1, 14]. Tumor cells were injected intraperitoneally into mice in all groups 7 days after the stress procedure began. A total of 10 mice per group were used. Starting 4 days after tumor cell injection, mice were treated with siRNA (control or MCP1-specific, 3.5 μg in DOPC every 3 days, intraperitoneally) for the duration of the experiments. Animals were sacrificed 28 days after tumor cell injection.

### Patient demographics

Following approval by the local institutional review boards, clinical data from primary epithelial ovarian cancer patients from four U.S. academic medical centers were analyzed for this study. Out of 537 initial patients, 462 patients had complete blood count from the time of diagnosis. Of these, 76 patients had available tissue samples from their initial debulking. Primary tumor samples from these patients were analyzed for CD68 expression. Patient and tumor characteristics are included in Supplementary Table 2.

### Immunohistochemistry

Formalin-fixed, paraffin-embedded human samples were stained for CD68. Slides were heated overnight at 65°C and then deparaffinized using graded xylenes. Antigen retrieval was performed using Citrate Buffer solution in a vegetable steamer for 40 min and followed by a 30-min cool-down. Endogenous peroxidase activity was blocked by incubation in 3% hydrogen peroxide (in methanol) for 12 min. The protein blocking solution was applied for 30 min using an Fc Receptor Blocker (Innovex Biosciences, Richmond, CA). Primary antibodies were applied at a dilution of 1:200 and incubated overnight in a humidity chamber at 4°C. Secondary antibodies were visualized using the MACH4+ polymer-linked horseradish peroxidase system (Biocare, Concord, CA). 3,3′-Diaminobenzidine was applied, and staining was monitored visually under a bright field microscope (~ 3–4 min). Counterstaining was performed using Gill's Hematoxylin for 10 sec, followed by washing with phosphate-buffered saline. Slides were dried and mounted using universal mounting medium and then scored by a board-certified pathologist. Counts were determined by the amount of CD68+ cells per field.

### Co-immunofluorescence staining for CD-14 and MCP-1

Paraffin-embedded samples from SKOV3ip1 tumor bearing mice were deparaffinized, permeabilized in cold methanol, incubated overnight with CD-14 and MCP1 antibody, stained with an anti-rabbit and anti-mouse secondary antibodies, respectively, secondary antibody conjugated to a Cy3 or Cy5 fluorochrome (Molecular Probes, Eugene, OR), and visualized with a confocal microscope.

### Statistical analysis

We compared continuous variables using either Student's t-test or analysis of variance and compared the categorical variables using the chi-squared test. When appropriate, we used a nonparametric test (Mann-Whitney test) to compare differences. The best cutoff for disease-specific survival was based on ROC curve analysis. MCP1 levels, CD68+ cells and monocyte levels were compared using spearman's correlation analysis. Survival curves were analyzed by the Kaplan-Meier method, and statistical significance was determined by the log-rank test. We considered *P* < 0.05 to be significant.

## SUPPLEMENTARY FIGURES AND TABLES


