# Supplementary material for: Adrenergic regulation of monocyte chemotactic protein 1 leads to enhanced macrophage recruitment and ovarian carcinoma growth
Source: Oncotarget. 2014 Dec 27;6(6):4266–73. doi: 10.18632/oncotarget.2887 (PMC4414188; doi:10.18632/oncotarget.2887)
Supplement: Supplementary file 1 [file oncotarget-06-4266-s001.pdf]

## SUPPLEMENTARY FIGURES AND TABLES

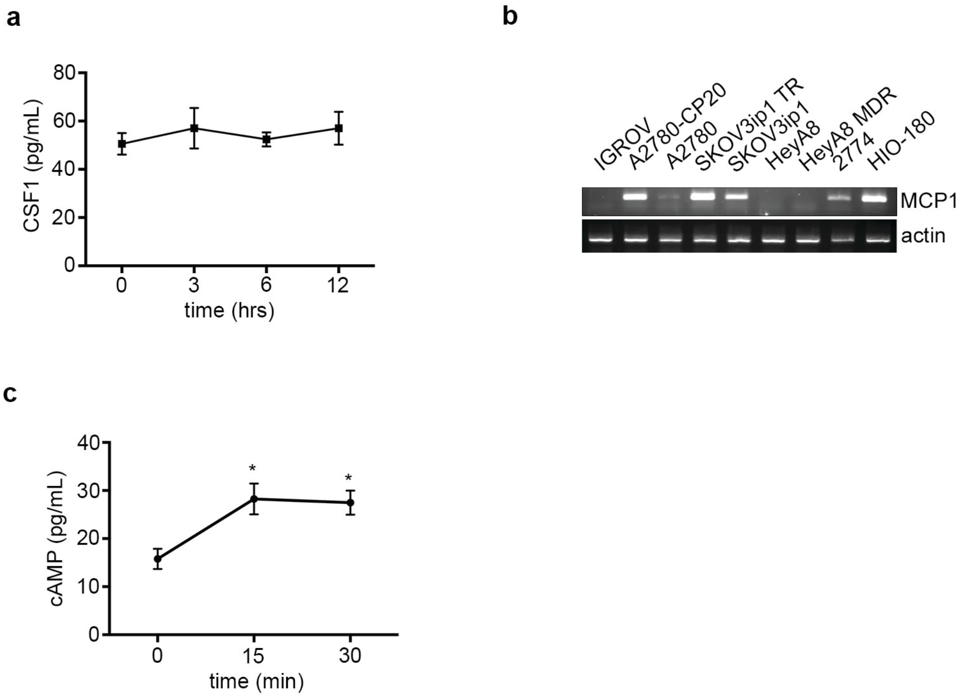


**Supplementary Figure 1: (a)** CSF1 protein levels on SKOV3ip1 cell supernatant after NE treatment. **(b)** MCP1 mRNA expression in a panel of ovarian cancer cells and a immortalized ovarian epithelial cell line (HIO-180). **(c)** cAMP levels after NE treatment on SKOV3ip1 cells.

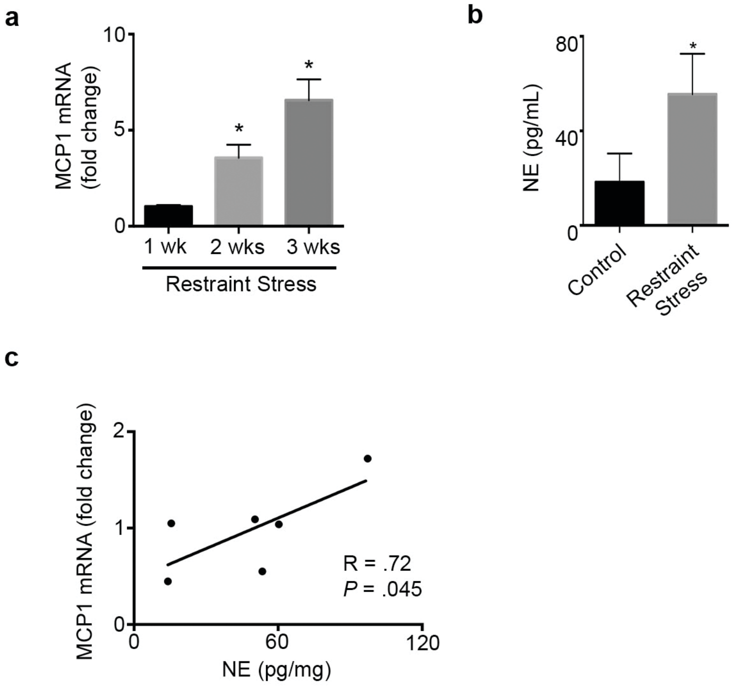


**Supplementary Figure 2: (a)** MCP1 mRNA levels in tumor samples from mice subjected to daily restraint stress. **(b)** Tumoral NE concentration from SKOV3ip1 tumor samples. **(c)** Correlation between tumoral NE levels and MCP1 gene levels on SKOV3ip1 tumor samples.

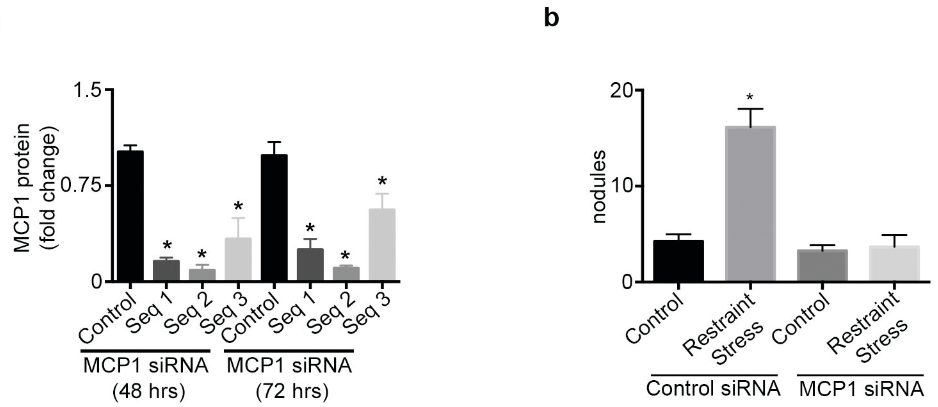


**Supplementary Figure 3: (a)** MCP1 protein concentration from SKOV3ip1 cells treated with three different MCP1 siRNA sequences. **(b)** Nodule counts in restraint stress orthotopic SKOV3ip1 model treated with control or hMCP1 siRNA-DOPC nanoparticles.

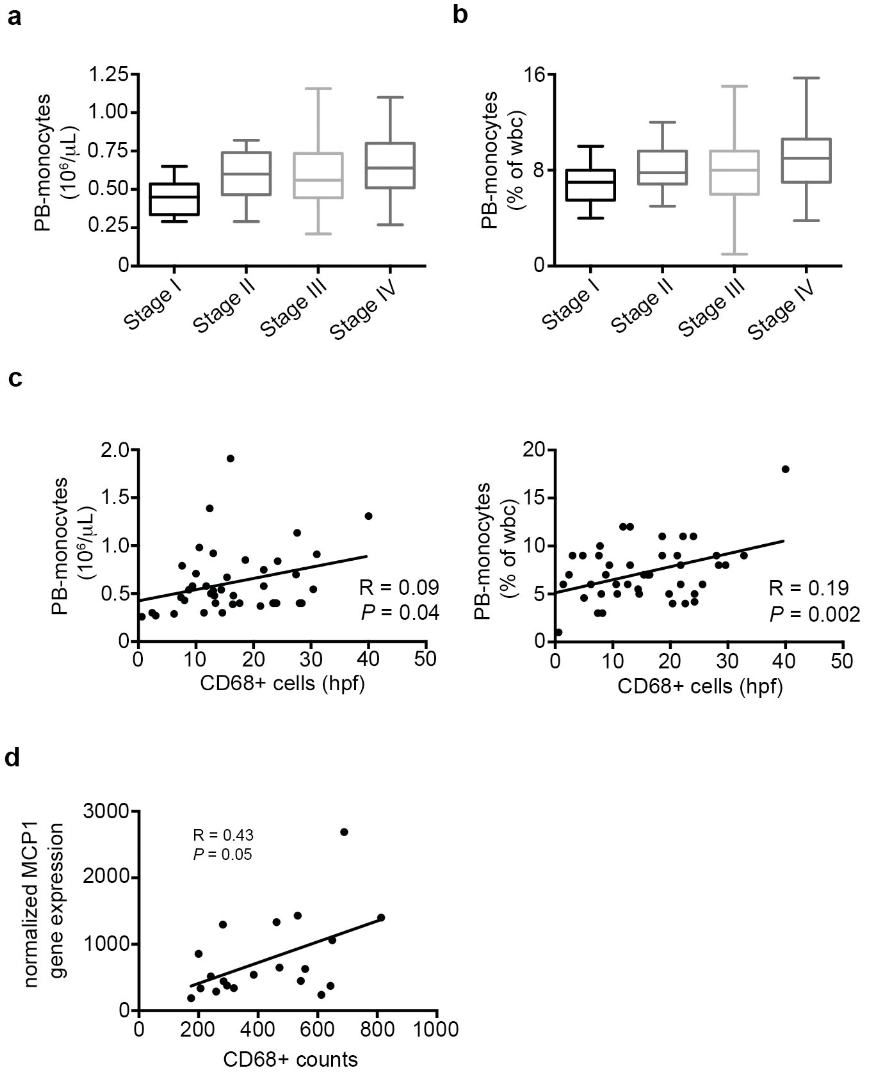


**Supplementary Figure 4: (a–b)** Box-plots showing the distribution of monocyte levels among ovarian cancer patients. **(c)** Linear regression comparing peripheral blood monocytes with tumoral macrophages. **(d)** Correlation between tumor associated macrophages and MCP1 gene levels on patient samples.

**Supplementary Table 1: Human cytokine/chemokine analysis of SKOV3ip1 cells treated with NE, Epi or Iso for 3 hrs (results shown as average among triplicates; pg/mL)**

| Protein Name | Control | Norepinephrine | Epinephrine | Isoprotenerol |
|---|---|---|---|---|
| EGF | 0 | 3.93 | 4.01 | 4.06 |
| Eotaxin | 1.67 | 0.87 | 4.51 | 4.7 |
| FGF-2 | 2.34 | 0 | 0 | 2.67 |
| Fit-3-Ligand | 0.13 | 0.9 | 0.13 | 0.63 |
| Fractalkine | 0 | 29.07 | 38.98 | 37.61 |
| G-CSF | 0 | 0 | 0 | 0 |
| GRO | 129.64 | 121.25 | 199.87 | 410.05 |
| INFa2 | 0 | 0 | 0 | 0 |
| INFg | 0 | 1.43 | 1.89 | 2.81 |
| IL-1a | 0 | 0 | 0 | 0 |
| IL-1b | 0 | 0.36 | 0 | 0.1 |
| IL-1ra | 0 | 0 | 0 | 0 |
| IL-2 | 0 | 0 | 0 | 0 |
| IL-3 | 0 | 0 | 0 | 0 |
| IL-4 | 0 | 0 | 0 | 0.31 |
| IL-5 | 0 | 0 | 0 | 0 |
| IL-6 | 3.63 | 139.02 | 94.67 | 153.66 |
| IL-7 | 0 | 3.1 | 4.03 | 3.88 |
| IL-8 | 0 | 143.09 | 136.92 | 163.35 |
| IL-9 | 0 | 0 | 0 | 0 |
| IL-10 | 0 | 0 | 0 | 0 |
| IL-12 (p40) | 0 | 1.075 | 1.41 | 1.59 |
| IL-12 (p70) | 0 | 0 | 0 | 0 |
| IL-13 | 0 | 0 | 0 | 0 |
| IL-15 | 0 | 0 | 0 | 0 |
| IL-17a | 0 | 0 | 0 | 0 |
| IP-10 | 0 | 0 | 0 | 0 |
| MCP-1 | 0 | 2.95 | 2.8 | 3.58 |
| MCP-3 | 2.666 | 2.66 | 3.338 | 3.14 |
| MDC | 0 | 0 | 0 | 0 |
| MIP-1a | 0 | 0 | 0 | 0 |
| MIP-1b | 0 | 0 | 0 | 0 |
| sCD40L | 0.02 | 0.98 | 0.49 | 0.02 |

(*Continued*)

| Protein Name | Control | Norepinephrine | Epinephrine | Isoprotenerol |
|---|---|---|---|---|
| SIL-2Ra | 0 | 0.7 | 0.9 | 0.59 |
| TGFa | 0 | 0 | 0 | 0 |
| TNFa | 0 | 0 | 0 | 0 |
| TNFb | 0 | 0.275 | 0.445 | 0.39 |
| VEGF | 0 | 26.44 | 83.93 | 50.99 |
| GM-CSF | 0 | 0 | 0 | 0 |

## Supplementary Table 2: Patient and tumor characteristics

| Variable | # (%) |
|---|---|
| Stage* | |
| I/II | 83 (15.45%) |
| III/IV | 454 (84.55%) |
| Grade** | |
| Low | 56 (10.43%) |
| High | 481 (89.57%) |
| Histology** | |
| Serous | 473 (88.08%) |
| Other | 64 (11.92%) |
| Cytoreduction*** | |
| Optimal | 322 (59.96%) |
| Suboptimal | 215 (40.04%) |
| Monocytes (absolute)**** | |
| Elevated | 200 (43.29%) |
| Monocytes (%)**** | |
| Elevated | 131 (28.30% |
| Beta-blocker Therapy***** | |
| Monocyte Cohort | 37 (13.65%) |
| Macrophage Cohort | 4 (6.67%) |

*as determined by the International Federation of Gynecology and Obstetrics staging system
**as determined by pathologic review of specimens
***optimal surgical cytoreduction defined as all disease nodules at completion of primary cytoreductive surgery being <1 cm
****Total number of patients is 462 for absolute monocyte and % monocytes of wbc due to limited patient records
*****Total number of patients is 271 for the monocyte cohort and 60 for macrophage cohort due to limited patient records
